# Recent advances in NIR-II fluorescence based theranostic approaches for glioma

**DOI:** 10.3389/fchem.2022.1054913

**Published:** 2022-11-04

**Authors:** Jiaying Li, Jue Ling, Chaoyi Yao

**Affiliations:** ^1^ Department of Nosocomial Infection Management, Nantong Third People’s Hospital Affiliated to Nantong University, Nantong, Jiangsu, China; ^2^ Key Laboratory of Neuroregeneration, Ministry of Education and Jiangsu Province, Co-innovation Center of Tissue Engineering and Nerve Injury Repair, Nantong University, Nantong, China; ^3^ School of Chemistry and Chemical Engineering, Queen’s University Belfast, Belfast, United Kingdom

**Keywords:** fluorescence imaging, NIR dyes, delivery system, photodynamic therapy, photothermal therapy, glioma

## Abstract

Gliomas are among the most common malignant tumors in the central nervous system and lead to poor life expectancy. However, the effective treatment of gliomas remains a considerable challenge. The recent development of near infrared (NIR) II (1000–1700 nm) theranostic agents has led to powerful strategies in diagnosis, targeted delivery of drugs, and accurate therapy. Because of the high capacity of NIR-II light in deep tissue penetration, improved spatiotemporal resolution can be achieved to facilitate the *in vivo* detection of gliomas *via* fluorescence imaging, and high contrast fluorescence imaging guided surgery can be realized. In addition to the precise imaging of tumors, drug delivery nano-platforms with NIR-II agents also allow the delivery process to be monitored in real-time. In addition, the combination of targeted drug delivery, photodynamic therapy, and photothermal therapy in the NIR region significantly improves the therapeutic effect against gliomas. Thus, this mini-review summarizes the recent developments in NIR-II fluorescence-based theranostic agents for glioma treatment.

## Introduction

Gliomas account for 27% of intracranial primary tumors and originate from glial precursor cells (Blümcke et al., 2001; Ostrom et al., 2020; Balana et al., 2022). With high morbidity and mortality rates, gliomas are one of the most common malignant tumors in the central nervous system (Wen and Kesari, 2008; Razpotnik et al., 2017). Currently, gliomas are classified into subtypes, such as glioblastomas, anaplastic gliomas, and oligodendrogliomas. Glioblastomas account for 57.7% of all gliomas (Sailor and Park, 2012; Wesseling and Capper, 2018; d’Angelo et al., 2019). Depending on the location of the primary tumor and degree of malignancy, the treatment of gliomas remains challenging and prognosis is poor. The 5-year survival rate of patients affected with glioblastoma is less than 5% (Khaddour et al., 2020). Like many other malignant cancers, the most common treatment strategy for brain tumor patients is still surgery. Computed tomography (CT), magnetic resonance imaging (MRI) and positron emission tomography (PET) are the most common imaging modalities for surgery preparations (Dasgupta and Haas-Kogan, 2013; McNamara et al., 2014; Silantyev et al., 2019). However, due to their disadvantages such as unfriendly to pregnant women, children, patients with metal implants and patients with claustrophobia, novel imaging strategies especially real time ones need to be developed to improve the complete resection of gliomas. Most brain tumors, especially glioblastoma (GBM), can infiltrate into normal brain tissues, making it almost impossible to distinguish the tumor margins during surgery. In this instance, other precisely tumor-targeting clinical therapeutic methods such as irradiation and chemotherapy usually would be applied combined with surgery to improve the therapeutic efficacy. Phototherapy can also be a complementary to the current clinical treatment for gliomas. However, most of phototherapy is focused on tackling skin-related diseases because of the tissue penetration problem of excitation light, in which near infrared (NIR) fluorescence can help since it has deep tissue penetration. The tight junctions (TJs) of endothelial cells between the central nervous system (CNS) and peripheral blood circulation form the blood brain barrier (BBB) which presents an obstacle to various macromolecule/nanoparticles as contrast agents or therapeutic drugs into the brain. Many novel NIR fluorescent systems have been designed to across BBB and cure glioma-related problems.

**FIGURE 1:**
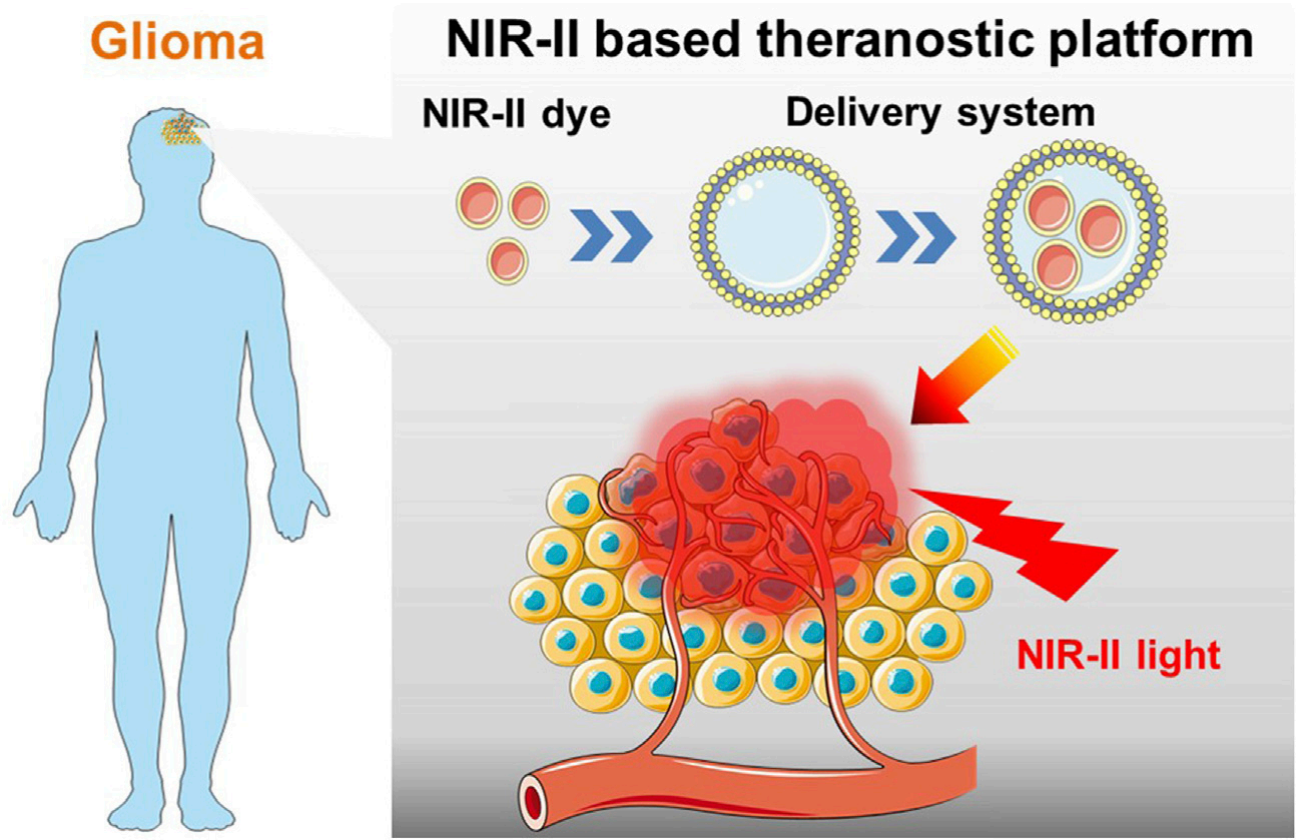
Schematic of NIR-II probes equipped with targeted drug delivery nano-platforms for precise diagnosis and effective phototherapy of glioma.

Fluorescence imaging offers an opportunity for the precise detection of primary tumors or metastasis (Guilbault, 2020; Ling et al., 2021). Methods, such as photoinduced electron transfer (Daly et al., 2015) and intramolecular charge transfer (Misra and Bhattacharyya, 2018) involve the fabrication of stimuli-responsive fluorescent moieties that can accomplish functions, such as sensing (Schäferling, 2012). Recent developments of fluorescent applications for use in the life sciences have revealed the power of fluorescence for tracking and analyzing biological molecules (Liu H. W. et al., 2018; Huang Z. et al., 2021; Wu et al., 2022), labelling and delivering drugs (Guo et al., 2018; Patsenker and Gellerman, 2020), photodynamic therapy (PDT) (Li L. et al., 2019; Cai and Liu, 2020; Tang et al., 2021), and photothermal therapy (PTT) (Bian et al., 2021). In the case of gliomas, fluorescence has garnered considerable attention for its applicability in tumor diagnosis and therapy. Several fluorophores have been applied to gliomas for imaging, drug delivery, and therapy (Ji et al., 2018; Mishchenko et al., 2020; Ghirardello et al., 2022; Xiang et al., 2022). However, the emissive range of these fluorophores is within the visible light spectrum (380–700 nm), which leads to poor tissue penetration owing to light absorption and scattering and autofluorescence. Considering this, novel molecules and materials that emit light in the near infrared (NIR) range (650–1700 nm), especially within NIR-II (1000–1700 nm), are required to realize *in vivo* fluorescence imaging, drug delivery, and efficient PDT and PTT therapies (Figure 1). Thus, this review focuses on recent research in NIR-II fluorescence for glioma treatment concerning three areas: imaging, drug delivery, and therapy.

## NIR-II fluorescence imaging

The NIR-II region (1000–1700 nm) has better spatiotemporal resolution and deeper tissue penetration than the NIR-I region (700–950 nm) and has attracted considerable interest from several research groups recently (Lu et al., 2020; Zhang et al., 2021). With the help of nanotechnology, through-skull fluorescence imaging in the NIR-IIb region (1500–1700 nm) has the potential to facilitate highly precise imaging-guided tumor surgery (Wang et al., 2022). The development of NIR-II fluorescent agents also significantly broadened the application scope for the fluorescence imaging of gliomas.

Based on their previous research (Liu et al., 2019), Li et al. investigated Er-based lanthanide down-conversion nanoparticles (DCNPs), which produce intense emissions in the NIR-IIb range (1500–1700 nm) and can be used for *in vivo* through-skull glioma imaging (Ren et al., 2020). A 0.9 nm NaYbF_4_ interlayer of this core-shell-shell NaErF_4_:Ce@NaYbF_4_(0.9 nm)@NaLuF_4_ material reflects the radiative energy of Er^3+^ and sequentially drives cross-relaxation *via* Ce^3+^ to maximize the transition to produce 1525 nm emissions. To gain biocompatibility, a novel dye-brush polymer was designed and synthesized to encapsulate the DCNPs, resulting in an average hydrodynamic size of 39.9 ± 2.8 nm. The dye-brush polymer absorbed at the same wavelength as the DCNPs (808 nm), which acted as energy harvesters, benefiting the energy cascaded down-conversion process to afford enhanced NIR-IIb fluorescence. The angiopep-2 peptide was chosen as the receptor for this system in combination with the dye-brush polymer-coated DCNPs because it could bind with overexpressed low-density lipoprotein receptor-related protein (LRP) on glioma cells. Because of the relatively large size of this nanoprobe, focused ultrasound sonication was used to control the opening of the blood brain barrier (BBB) to deliver the nanoprobe. Experiments in mice revealed that this DCNP-based nanoprobe was capable of performing the through-skull fluorescence imaging of gliomas. This method is comparable with T2-weighted MRI and can probably be used for intraoperative visualization. To improve the BBB permeability, Liu et al. coated a brain tumor cell membrane with Er-based lanthanide doped nanoparticles to form an NIR-IIb fluorescence imaging agent that targets glioma (Wang et al., 2022b). This nanoparticle, NaYbF_4_:Gd,Er,Ce@NaYF_4_, has a diameter of approximately 50 nm and is capable of coating human glioblastoma cell membrane vesicles after modification with phosphatidylcholine to increase the water dispersibility. Animal experiments revealed that this system has good immune escape, BBB crossing abilities, and homologous targeting abilities. Angiopep-2 is another glioma-targeting peptide that has a high binding affinity for low-density lipoprotein receptor-related protein-1. Conjugation with NIR-II fluorescent Ag_2_S quantum dots allows for the *in vivo* visualization of gliomas (Xu et al., 2018).

Similar to lanthanide-based nanoparticles and quantum dots, molecular fluorophore-based nanoparticles can also emit in the NIR-II region as nanoprobes to aid in fluorescence-image guided surgery (FIGS). Hong et al. developed a polymethine-based nanoprobe that both absorbs and emits in the NIR-II region. The NIR-II dye is designed to substitute the pyrrole of the indocyanine green (ICG) into thiopyrylium to induce NIR-II excitation and emission (Ding B. et al., 2019). Its nanoparticle version was tested in mice for tumorous microvasculature imaging. The nanoprobe conjugates with cyclic arginine-glycine-aspartate motif (cRGD) *via* the polyethylene glycol (PEG) chain by a click reaction, and has the potential to realize glioma fluorescence imaging. Instead of using a benzo-bis(1,2,5-thiadiazole) acceptor-centered fluorophore (Sun et al., 2016), Xiao et al. discovered the thiophenthiadiazole-derived fluorophore, which can self-assemble into spherical or vesicular micelles (50–200 nm) after conjugating with the PEG chain through carboxylate groups (Li et al., 2022). These micelles can target solid tumors through the enhanced permeability and retention (EPR) effect. *In vivo* experiments in mice reveal that this nanoprobe has little immunogenicity, low binding affinity, ultralong blood circulation, and high contrast, which make it a promising novel tool for NIR-II FIGS.

## NIR-II fluorescence-based drug delivery

The study and design of artificial delivery routes allows researchers to investigate treatments for critical diseases. Drug delivery systems are used to deliver drugs to the desired tissues and organs, and allow for controllable drug release by multifunctional drug carriers (Li N. et al., 2019; Zhang Y. et al., 2022). Drug delivery can solve many existing problems that limit the use of therapeutic drugs, such as poor solubility, lack of biodistribution and bioavailability, low selectivity, and side effects (Liu et al., 2015; Liu D. et al., 2018; Huang X. et al., 2021; Wei et al., 2021; Zhou et al., 2021; Kalot et al., 2022). Recently, NIR fluorophore-functionalized delivery systems targeting gliomas have been developed that allow the visualization of the delivery process and monitoring of drug behavior.

Grossman et al. tested lipid-based microbubbles (MBs), which are mature ultrasound contrast agents, as carriers (Paradossi et al., 2019). The glioma-targeting peptide, cRGD, which contains a cysteine moiety, was coupled to the MB through a maleimide group together with ICG. After engineering MBs with the dye moiety, ICG, and targeting moieties, the material was further tested in mice and demonstrated accumulation in gliomas without cytotoxicity at a dose of 253 mg/kg. MBs can carry some therapeutic agents, which means they have the potential to become the carriers for glioma drug delivery with coupled with NIR fluorescence imaging. Kim et al. further tested the drug delivery ability of the MBs. As ultrasound waves can oscillate and destroy the MBs, Kim et al. established an MB-based drug delivery system to cross the BBB, which typically limits the delivery of large molecules (400–500 g/mol) (Ha et al., 2019). In their system, Cy5.5 doped albumin nanoparticles decorated the surface of an NHS-functionalized MB. In this case, Cy5.5 was the NIR fluorophore and albumin was the glioma-targeting agent. By applying ultrasound (0.1 W/cm^2^, 10% duty cycle, 5 min), NIR fluorescent images of these engineered MBs in mouse brain reveal a strong fluorescence accumulation around gliomas compared to the no ultrasound group. This indicates that the BBB was penetrated efficiently, and the NIR fluorophores acted as imaging agents and drug carriers.

Instead of MBs, Shi et al. applied a similar strategy to liposomes, and investigated the precise delivery of (–)-gossypol (AT-101) as a drug for the treatment of glioma (Liu H. et al., 2022). The application of AT-101 is limited because of its hydrophobicity and non-tumor specific biodistribution. In their system, the cRGD (glioma targeting agent) and DiR (NIR fluorescence imaging agent) together with (-)-gossypol are coated on the surface of liposomes. NIR fluorescence enabled the researchers to monitor the release of (-)-gossypol *in vivo*.

The BBB limits the delivery of many therapeutic agents to glioma. Wang et al. adopted an *in situ* self-assembly strategy using nanotherapeutic agents to penetrate the BBB (Gao et al., 2020). Their system is based on RGD peptide-modified bisulfite-zinc(II)-dipicolylamine-Arg-Gly-Asp (Bis(DPA-Zn)-RGD), which is positively charged and is capable of targeting tumors. After the accumulation of Bis(DPA-Zn)-RGD in glioma, ultrasmall Au-ICG nanoparticles (∼7 nm) cross the BBB and self-assemble with Bis(DPA-Zn)-RGD.

Instead of tagging a carrier with an NIR fluorescent agent, Zhou et al. discovered an NIR fluorescent agent that could act as a carrier (Li et al., 2020). They discovered a large amino acid-mimicking material, functionalized carbon quantum dots (CQDs). The edges of the CQDs are functionalized with paired *a*-carboxyl and amino groups, which trigger multivalent interactions with the large neutral amino acid transporter 1 (LAT1). As LAT1 is the overexpressed carrier transporter in cancer cells, these CQDs can target glioma cells selectively. More importantly, a large *p*-conjugating system enabled the CQDs to transfer drugs through π–π interactions and emit NIR fluorescence as an imaging agent. The material demonstrated efficient BBB penetration during *in vivo* experiments and could act as both an NIR fluorescence imaging tool and a drug delivery carrier.

Zhang et al. developed an NIR-II fluorescent nano carriers with YVO_4_:Nd^3+^ particles as the cores and MnO_2_ nanosheets as the shells (Lv et al., 2022a). They tested the drug delivery ability of this material in glioma by loading the sonosensitizer, hematoporphyrin monomethyl ether, and glioma targeting agent, lactoferrin, onto the surface. The NIR-II fluorescent nanoparticles acted as carriers, but also enhanced the effect of the sonodynamic therapy (SDT) by providing O_2_ to the tumor from the MnO_2_ shell to appease the hypoxia caused by SDT. Animal experiments revealed that this material efficiently facilitated the treatment of glioma by SDT.

## NIR-II based phototherapy

There are many NIR-II fluorescent agents available, not only for efficient fluorescence imaging, but also for phototherapy (Hu et al., 2021; Liu et al., 2021; Liu Y. et al., 2022; Zhang L. et al., 2022; Du et al., 2022; Lee et al., 2022; Nirmal et al., 2022; Shi et al., 2022; Sun et al., 2022). The level of deep tissue penetration using the NIR-II window significantly expands the application scope of phototherapy. Zhang et al. developed a graphene quantum dot (∼5 nm) doped with N and B as an NIR-II fluorescence imaging and photothermal therapeutic agent (Wang et al., 2019), respectively. This graphene quantum dot exhibits good photostability and excitation wavelength-dependent emissions in the NIR-II window. The NIR-II emissions attributed to the N and B doping induced a local distortion of electron energy, which created additional energy gaps, and a vacancy deficiency, which redshifted the emission peak. *In vivo* experiments revealed that this material is efficient for the NIR-II fluorescence imaging of internal organs and blood vessels. *In vitro* experiments on glioma cells revealed that the material has a high potential for application in PTT.

Kim et al. combined chemotherapeutic organoplatinum (II) metallacycles and NIR-II fluorophores with an FDA-approved amphiphilic polymer of poly (ethyleneoxide)-poly (propylene oxide)-poly (ethylene oxide) (Pluronic F127) to form a nanotheranostic agent (∼110 nm) (Ding F. et al., 2019). The polymer encapsulating two components improves the photostability, fluorescence tissue penetration, solubility, and biocompatibility as well as accumulation in tumor sites *via* the EPR effect. The glioma treatment was tested *in vivo*. Other novel materials can be combined to make nanotheranostic systems. Tian et al. developed a system suitable for both MRI and NIR-II fluorescence imaging by loading an Fe-based metal-organic framework with an NIR-II molecular fluorophore and AE105 peptide designed to target overexpressed urokinase plasminogen activator receptor on glioblastoma. *In vivo* studies reveal that this system can realize efficient PTT and achieve the successful ablation of tumors.

Conjugated polymers are promising materials for NIR-II fluorescence because the gap between the highest occupied molecular orbital (HOMO) and lowest unoccupied molecular orbital (LUMO) can be reduced. However, aggregation-induced quenching limits their applicability to the construction of nano therapeutic and imaging agents. Aggregation-induced emission (AIE) materials can solve this problem. AIE polymer-based nanomaterials with a high quantum yield (∼7.9% in water) have been developed by Deng et al. (2020). The AIE Polymer is encapsulated by lipids and then loaded with a nature killer cell membrane, which can target tumor cells and unzip the tight junction structure of BBB. *In vivo* animal experiments revealed that this nanomaterial is efficacious for NIR-II fluorescent through-skull imaging and PTT.

Previous AIE nanomaterials emit at approximately 1000 nm. Tang et al. pushed the emission wavelength to 1550 nm, which falls within the NIR-IIb window. To decrease the gap between HOMO and LUMO and gain a large redshift emission, a D-π-A AIE fluorophore was synthesized with planar blocks associated with a twisted skeleton to simultaneously achieve high absorbance and quantum yield (Wang et al., 2022a). The tail emission extends to 1550 nm, which allows NIR-IIb fluorescence imaging *in vivo*, and the D-π-A AIE fluorophore self-assembles into nano aggregates and is further loaded with the brain-targeting apolipoprotein E peptide. This nanoparticle has good BBB penetration and high PTT efficiency and prolongs the lifespan of mice suffering from orthotopic glioblastoma.

## Conclusion and future perspectives

In this review, we have given some representative NIR fluorescent examples for solving problems of glioma diseases in the past 5 years. This review’s focus is on imaging, delivery and therapy. In the fluorescence imaging part, intraoperative glioma visualization with NIR-fluorescence has been highlighted since clinical trials have already been conducted. Application of fluorescence imaging of gliomas in the NIR region has flourished by significantly increasing the spatial and temporal resolution. However, it is noticed that NIR fluorescence as an information processing tool has been underestimated. Very few NIR fluorescent materials involving molecular logic gate concepts have been studied in gliomas. For example, most of the fluorescent probes have been designed as fluorophore at delivery ‘cargo’. In this way, the sensitivity will depend on the efficiency of glioma targeting. In the delivery part, NIR has been widely used as the monitoring tool in drug delivery and shows great efficacy in animal experiments. But this function is realized commonly by separately including fluorophores. The NIR-fluorescent delivery ‘cargo’ would be a promising area to develop. Phototherapy has the non-invasive advantage and minimal side effects which makes it ideal for glioma treatment. The NIR window improves the phototherapy’s efficacy by permitting deep tissue penetration. The NIR-II window even gives through-skull penetration in mouse experiments. Many novel materials have been designed to accomplish more red-shifted emissions.

To date, significant progress has been made towards the *in vivo* diagnosis of gliomas at the preoperative stage using fluorescence imaging in the NIR-II region. However, several challenges remain, such as the accurate detection of cancer metastasis at the preoperative or even intraoperative stage and improvement of the spatial and temporal resolution. Although the NIR-II spectra is available for through-skull penetration *in vivo*, the poor capacity of NIR-II nano-systems for crossing the BBB significantly limits the imaging contrast and therapeutic efficacy for glioma detection (Zhang H. et al., 2022; Lv et al., 2022b; Men et al., 2022). By equipping fluorescent probes with targeted delivery systems, the accumulation of NIR agents at glioma tumor sites can be improved. Thus, further investigation on the pathology of gliomas is necessary before new target sites can be found to enhance the targeting efficiency. Furthermore, the biocompability and biodistribution of NIR-II nano-systems should be evaluated and optimized to ensure their safety in clinical studies.
